# Supplementary material for: MetaRibo-Seq measures translation in microbiomes
Source: Nat Commun. 2020 Jun 29;11:3268. doi: 10.1038/s41467-020-17081-z (PMC7324362; doi:10.1038/s41467-020-17081-z)
Supplement: Supplementary file 10 — Supplementary Data 7 [file 41467_2020_17081_MOESM10_ESM.zip › File2/Confidence_High_Taxonomy/111845_out.krona.html]

Javascript must be enabled to view this page.

members
magnitude
magnitudeUnassigned
count
unassigned
taxon
rank

111845\_out

6

superkingdom
2
6

6
1239
phylum

class
186801
6

order
6
186802

family
6
186803

genus
33042
6

1
2293088

SRS147139\_contig\_number\_35660
species

species

SRS098073\_contig\_number\_17859
2293087
1

1
2049024

SRS1055022\_contig\_number\_451
species

3
33043

SRS015431\_contig\_number\_14892SRS016132\_contig\_number\_5177SRS049446\_contig\_number\_1136
species
